# The Use of Image-Spectroscopy Technology as a Diagnostic Method for Seed Health Testing and Variety Identification

**DOI:** 10.1371/journal.pone.0152011

**Published:** 2016-03-24

**Authors:** Martina Vrešak, Merete Halkjaer Olesen, René Gislum, Franc Bavec, Johannes Ravn Jørgensen

**Affiliations:** 1 Faculty of Agriculture and Life Sciences, Institute for Organic Farming, University of Maribor, Pivola, Hoce, Slovenia; 2 Faculty of Science and Technology, Department of Agroecology, Aarhus University, Forsøgsvej, Slagelse, Denmark; College of Agricultural Sciences, UNITED STATES

## Abstract

Application of rapid and time-efficient health diagnostic and identification technology in the seed industry chain could accelerate required analysis, characteristic description and also ultimately availability of new desired varieties. The aim of the study was to evaluate the potential of multispectral imaging and single kernel near-infrared spectroscopy (SKNIR) for determination of seed health and variety separation of winter wheat (*Triticum aestivum* L.) and winter triticale (*Triticosecale* Wittm. & Camus). The analysis, carried out in autumn 2013 at AU-Flakkebjerg, Denmark, included nine winter triticale varieties and 27 wheat varieties provided by the Faculty of Agriculture and Life Sciences Maribor, Slovenia. *Fusarium* sp. and black point disease-infected parts of the seed surface could successfully be distinguished from uninfected parts with use of a multispectral imaging device (405–970 nm wavelengths). SKNIR was applied in this research to differentiate all 36 involved varieties based on spectral differences due to variation in the chemical composition. The study produced an interesting result of successful distinguishing between the infected and uninfected parts of the seed surface. Furthermore, the study was able to distinguish between varieties. Together these components could be used in further studies for the development of a sorting model by combining data from multispectral imaging and SKNIR for identifying disease(s) and varieties.

## Introduction

A main issue in the production and processing of wheat is infection by the plant pathogen *Fusarium* sp., which decreases yield and seed quality (owing to contamination with mycotoxins—trichothecenes) [1, 2]. Infected kernels may have the same weight and size as non-infected kernels, and it is therefore difficult to separate infected kernels from uninfected with seed conditioning equipment [1]. With hyper-spectral imaging in the 400–1000 nm wavelength range, *Fusarium* damage on maize [3] and wheat seeds [4, 5] has been successfully detected, but the high cost of cameras and large amounts of data demands for rapid data processing are limiting factors for commercial use of this method. A novel development within multispectral imaging has been seed health testing on spinach seeds in which seeds, infected with *Fusarium* sp. and *Alternaria alternate*, could be distinguished [6]. The use of SKNIR to find similarities and dissimilarities between different breeding lines of agricultural crops is known [7] but has not been widely implemented in practical breeding.

Separation of varieties and determination of their properties due to DUS standards (Distinctness, Uniformity, Stability) is significant for variety registration, plant breeders rights (Intellectual Property Rights) [8], as well as for development of new varieties and market, due to the large number of varieties suitable for different growing requirements and end use. Meeting the market requirements of varietal purity was highlighted by Shrestha et al. [8] and Zhang et al. [9] as a serious issue faced by the actors in the whole seed industrial chain (breeders, farmers, processors, traders, consumers). As characterization of varieties and identification of desired plant phenotype are time-consuming, expensive and labor-intensive, an advanced image-spectroscopy device would be therefore highly applicable in the seed industry [10]. NIR spectroscopy and imaging have been developed and introduced to classify and compare wheat varieties. Furthermore, these methods reduced human error, saved money and time and also provided more reliable and replicable results [7, 11, 12, 13].

A high-speed color image sorting device (from the National Manufacturing Co., Lincoln, Nebraska) is already commercially used by wheat breeders for removing impurity, identifying seeds with FHB (Fusarium Head Blight) damage and distinguishing kernel color [13]. On the other hand, the device is costly and based only on visible light. Pearson et al. [13] exposed the need for an affordable instrument that could separate kernels on both a visual (VIS) and a near-infrared (NIR) basis for quality as well as healthy seed status and which could accelerate variety development. Breeders are constantly struggling to gain seeds with special properties in order to improve the quality of the present lines [14]. Therefore, this highlights multispectral imaging and SKNIR as a non-destructive, rapid method with the possibility of analyzing varieties at single seed level according to morphological and biochemical traits.

The main objective of the research was the detection of the *Fusarium* sp. infection among winter wheat and triticale varieties. The second objective was to distinguish between varieties. Hypothesis of this study was that a combination of these two technologies would make it possible to develop a high-speed and low-cost seed sorting model regarding interior and surface seed characteristics as well as their health condition.

## Materials and Methods

### Seed materials

Twenty-seven winter wheat (*Triticum aestivum* L.) varieties and nine triticale (*Triticosecale* Wittm. & Camus) varieties were used in Multispectral and SKNIR analyses. A field experiment for selection of the most resistant varieties had been designed under conventional cropping practice (with 4 replications) in which each of the 36 treated varieties also had an untreated control set. The seed infection/the infection of the seeds, *Fusarium* sp., was due to a natural/field rate of infestation, which is contrary to reports of other authors according to which seed were artificial inoculated [2, 4, 6].

From each treated and untreated control variety, twenty-four seeds were separated for identification of diseases (multispectral imaging and seed health test). Of these, some were visually healthy seeds and some showed signs of infection (red color or spots on the surface, black points, damage epidermis).

### Multispectral imaging

The methodology developed by Olesen et al. [6] was used as a reference for capture of multispectral images. All digital images were captured with a Videometer Lab instrument (Videometer A/S, Hørsholm, Denmark), shown on S1 Fig, where each obtained image contained a petri dish with twelve selected seeds and two images were captured per variety. The multispectral images of 1,280 x 960 pixels were captured at 19 different spectral bands from VIS to NIR wavelengths (375 nm—970 nm).

### Seed health test and microscopy

After capturing, the seeds were incubated according to an ISTA (International Seed Testing Association) blotter test [15] as a control set for multispectral analysis. Seeds were first placed on micro plates (12×8) and soaked with deionized water overnight. Next day, micro plates with seeds were drained and placed for 24 hours in the freezer to kill the embryos. Afterwards, seeds were placed in the petri dish on the filter paper and moistened with 4 ml of deionized water, then closed with parafilm paper. Furthermore, this was placed in the dark room for seven days at 22°C with a 12 h-12 h light cycle with black light (Narva LT 18 W/073) during the night and cool-white fluorescent light (Philips TL18W/79) during the day. After a week, microscopic observations for determination of seedborne pathogens followed, according to Mathur and Kongsdal [16].

### Multispectral data analysis

Videometer software version 1.6 (Videometer A/S, Hørsholm, Denmark), using normalized canonic discrimination analysis (nCDA) with all 19 bands, was used for data transformation in the multispectral images analysis. CDA is defined and known as supervised Fishers linear classifier in the way to minimize the calculated Jeffries-Matusita distance between observations within the group and maximize distance between the known groups in tested samples (groups are: 36 different varieties or/and infected and uninfected parts of the seeds) [17]. However, as only the seeds within the images were of interest for analysis, background segmentation from the seeds was performed.

**Fig 1:**
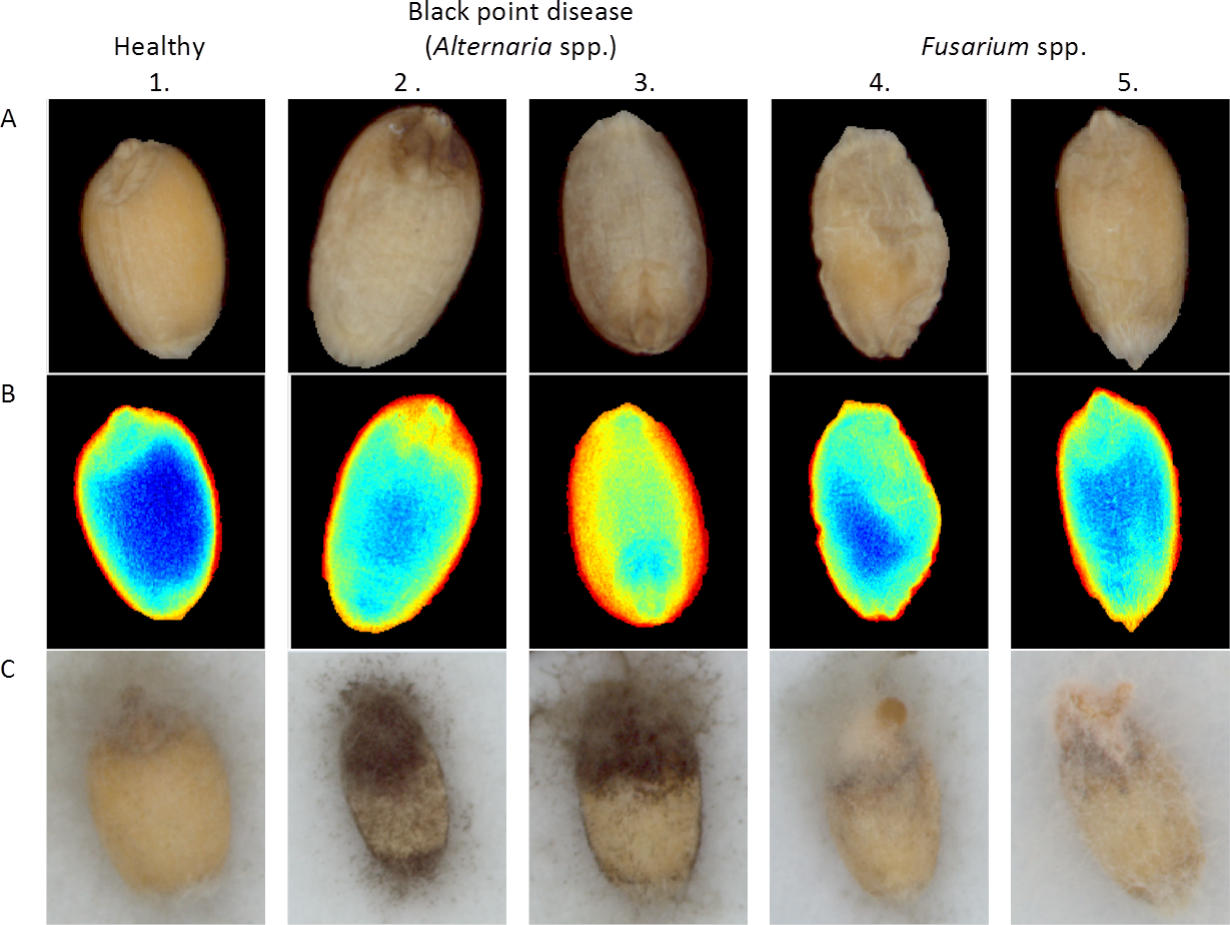
Image comparison of healthy seeds with infected seeds. Seeds on image were infected by black point disease (*Alternaria* sp.) and *Fusarium* sp. (A) RGB-captured images. (B) nCDA-transformed images. (C) RGB images after seed incubation.

Further data extraction was carried out on the infected seed part of the multispectral image called ROI -"Region Of Interest”. At first, ROIs were marked according to microscopic observations as well as based on the visual signs of infection/unification on dry seeds and then colored with different layers (yellow uninfected, red *Fusarium*-infected and green black point disease parts, which are caused by different fungi, like *Alternaria* sp., *Cladosporium* sp., etc.) to identify the groups and calculation of nCDA transformation. Afterwards, nCDA transformation was implemented on the rest of the images and examined to determine if the coloring corresponded to the microscope observation date (example presented on Fig 1). For visualization of data, a ROIs histogram was applied to illustrated differences in reflections intensity between identified groups.

For differentiation of varieties, the blob tool database was adjusted. At first background segmentation was implemented and afterwards each seed variety was colored with a different layer mask to identify them as different groups. Next, various multispectral imaging (MSI) nCDA transformation features were developed and employed together with different color, texture and shape features (area, length, width, roundness, intensity) of each seed to obtain the best variety discrimination in the score plot. The extracted features were used in a *k*-nearest neighbor (*k*-NN) based algorithm in order to differ and correlate the different varieties in a Confusion matrix. The *k*-NN algorithm is included in the Classification Development Tool (CDT) of the Videometer software.

### SKNIR (Single Kernel Near-InfraRed spectroscopy)

Seed also carry relevant information under the surface tissue which cannot be detected with multispectral imaging. Therefore, SKNIR was acquired from 900 to 1600 nm using a FT-NIR analyzer (Q-Interline A/S QFAflex 600; Tølløse, Denmark), shown on S2 Fig, to separate varieties based on information obtained from under the seed surface. Thirty seeds were randomly selected from each variety (15 treated and 15 from an untreated control set) to test a method for distinguishing different varieties of winter wheat and triticale. A principal component analysis (PCA) was applied after preprocessing the spectra in order to find similarities or dissimilarities between species.

## Results and Discussion

### Seed health test

In total 1,728 seeds (from 36 varieties) were included and microscopically evaluated for fungal infection. Due to the use of naturally infected seeds on the field, most of the seeds contained multiple infections. Therefore, it was difficult to find completely healthy seeds or seeds infected by just one disease. Of the seeds evaluated 60% was infected by *Alternaria* sp., and *Fusarium* sp. could be detected on 672 seeds. *F*. *graminearum*, *F*. *avanaceum* and *F*. *poe* were the three *Fusarium* species that were most frequently determined.

**Fig 2:**
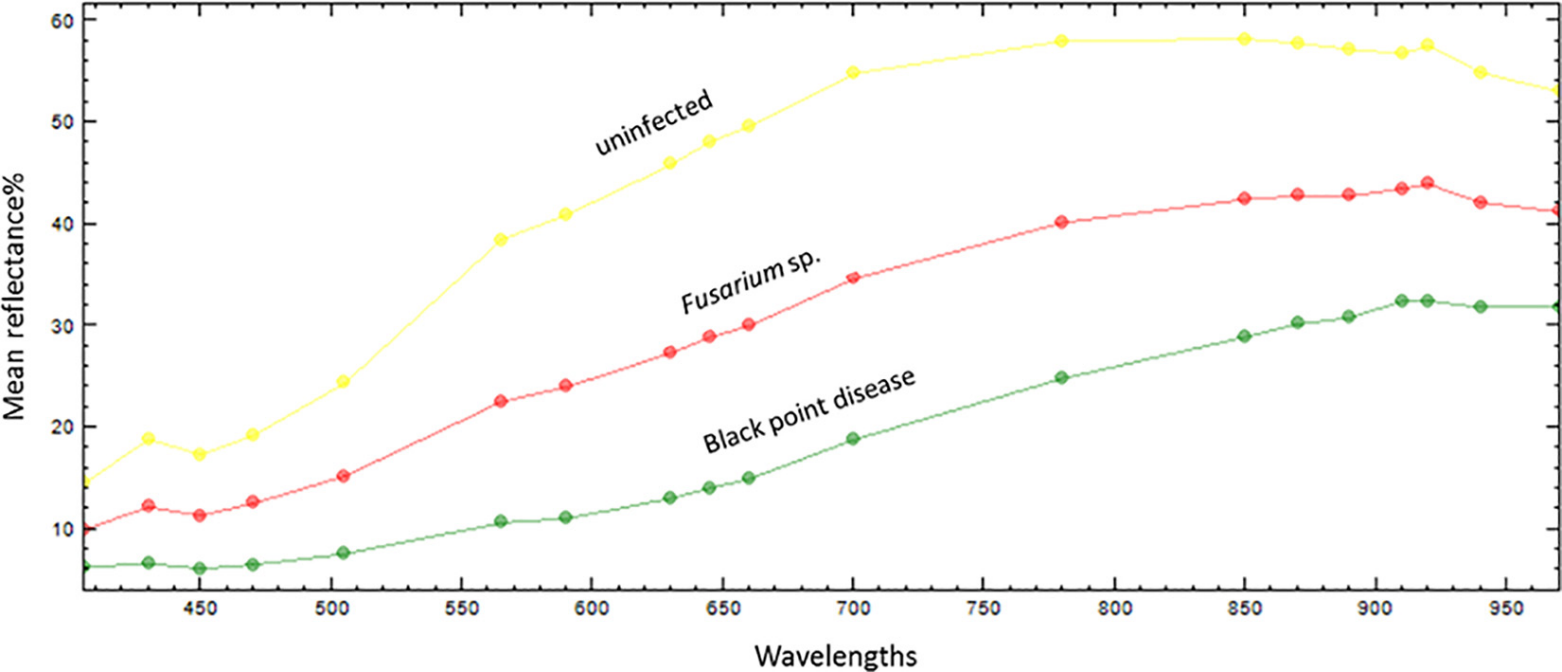
Reflection intensity between different infection/unifications. Separation spectrum of infected and uninfected ROI parts of the seeds regarding mean reflection intensity at different wavelengths.

The feature for distinguishing infected parts of seeds from uninfected ones was reflection intensity of pixels in each ROI, which was averaged to produce a mean value for each band and illustrated in a spectral curve diagram (Fig 2). At that point it was already possible to demonstrate that with multispectral imaging, distinctions can be made between the uninfected and infected parts of the seeds with *Fusarium* sp. and black point disease infection (in this study case: *Alternaria* sp.), which is clearly demonstrated also in Fig 1. Row A (in Fig 1) is performing traditional color (RGB) captured images of individual dry seeds captured before incubation; the following row B shows implementation of nCDA transformed on images to distinguish infected and uninfected seed parts (dark blue coloring for uninfected parts, orange color black point disease infection (in this study case: *Alternania* sp.) and light blue color for parts infected with *Fusarium* sp.), and in row C RGB images are captured after seed incubation, which were used for confirmation of correct dividing of infected and uninfected parts by nCDA transformation. The highlight of separation was linked to detected infection on the seeds parts that visually did not differ from uninfected ones (third and fifth column in Fig 1).

The uninfected ROI parts had the highest mean reflection intensity compared to *Fusarium* sp. and black point disease infection (Fig 2). At the 590–890 nm region, the mean value of reflection intensity between different infection/unifications was the largest. In the same spectral region Shahin et al. [5] reported that with use of hyperspectral imaging and the PLS regression model it was possible to extract four significant wavelengths (494, 578, 639 and 678 nm) for *Fusarium* seed damage estimation with overall 90% accuracy.

### Variety separation with multispectral imaging

**Fig 3:**
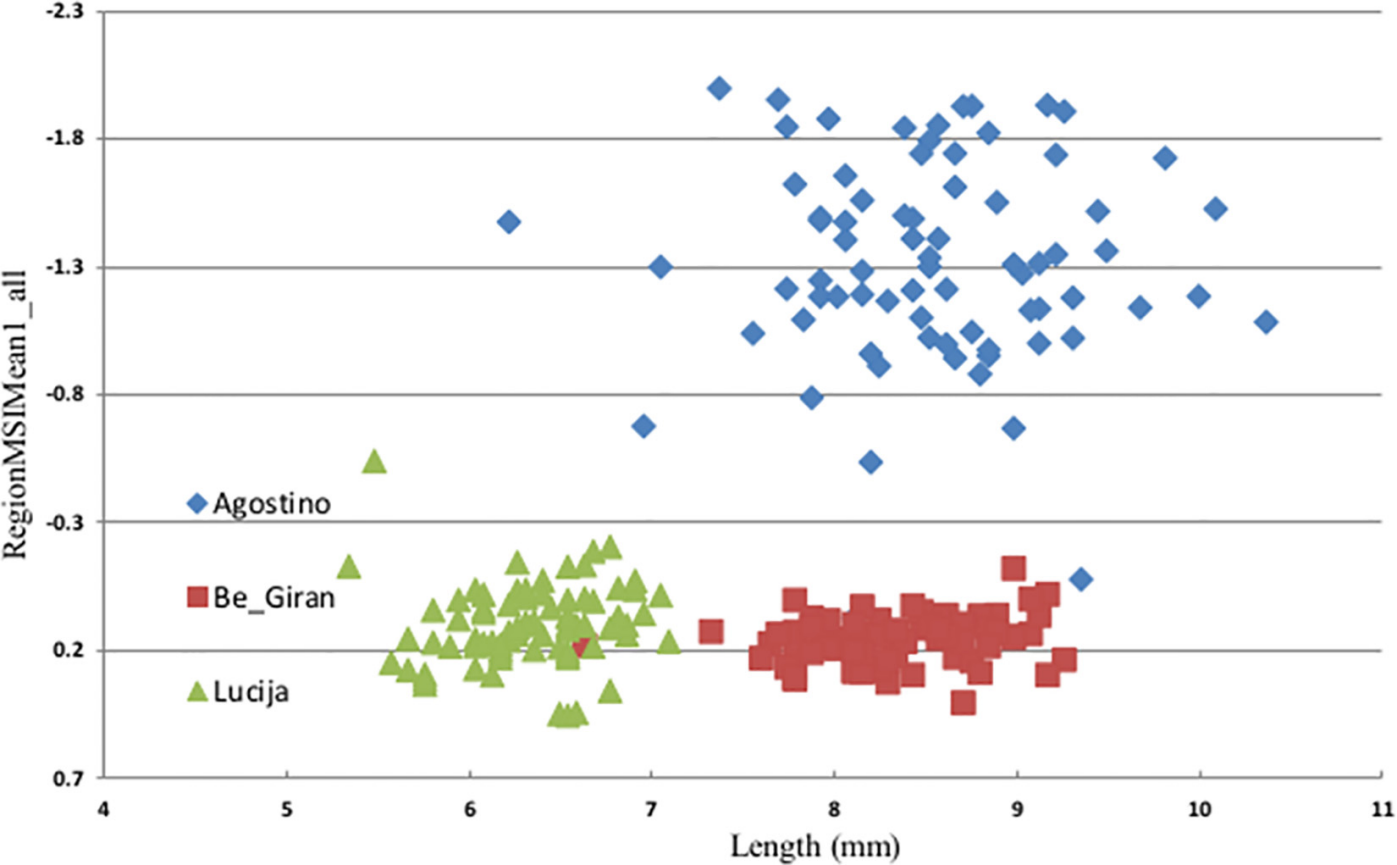
Blob tool database report of varieties separation regarding species, color and size of seeds. Varieties were separated by Region MSIMean1_all (nCDA created feature) and Length (mm). Agostino (green) is a triticale variety with long green seeds; Be_Giran is representing triticale varieties with long yellowish seeds and Lucija a group of winter wheat varieties with small yellowish seeds.

The varieties were classified according to color differences (green/yellowish) and size (long/small). Later on, when the sample numbers were deciphered, it was obvious that varieties were sorted not just according to color and size but also according to species (winter wheat/triticale), which divided varieties into three groups (example with three representative varieties in Fig 3). The results highlighted separation, but later on shape and color feature did not give any more desired separation for sorting varieties as individuals. Shrestha et al. [8] exposed the same issue in tomato varieties discrimination.

**Fig 4:**
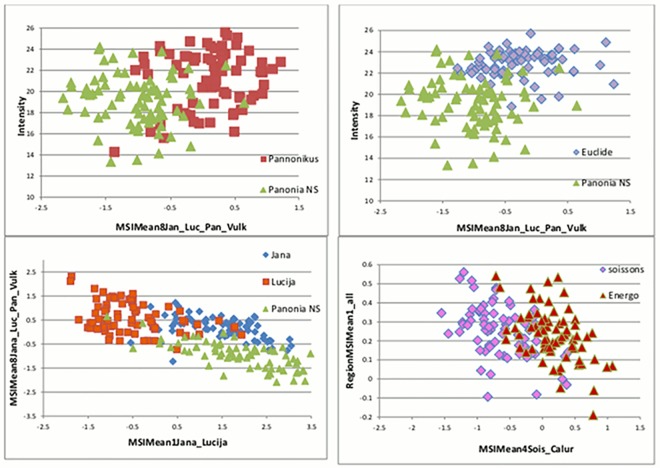
Example of successful separation of winter wheat. Varieties were separated by intensity and nCDA-created features from multispectral imaging (MSIMean): Pannonikus (red square), Panonic NS (green), Jana (blue), Lucija (orange), Euclide (violet), Soissons (pink), Energo (red triangle).

Therefore, for improved variety separation within this tree groups, selection of different variety combinations to create more pairwise nCDA transformation features (RegionMSImean for comparing all varieties or varieties in pairs) followed. There were few varieties, for example winter wheat varieties with short yellowish seeds: ‘Pannonikus’, ‘Panonic NS’, ‘Jana’, ‘Lucija’, ‘Euclide’, ‘Soissons’, ‘Energo’ on Fig 4, which could be separated easily among themselves when RegionMSImean in combination with intensity feature was employed. At the same time, there were a lot of varieties that were impossible to distinguish from others (in the same group) no matter what/which kind of combination of features was applied in the blob plot. The reason could be a common genetic background and large surface similarity (color/texture/chemical compositions). To get an overview of separation, data were further handled in Excel (results draft on Table 1).

**Table 1:** Confusion matrix of varieties and groups of varieties.

	Predicted						
Reference	Agostion	Calorius	Jana	Panonica NS	Lucija	Group 1	Group 2
Agostion	97.4	0.0	0.0	0.0	0.0	0.0	2.6
Calorius	0.0	67.1	0.0	0.0	0.0	0.0	32.9
Jana	0.0	0.0	39.5	0.0	0.0	53.9	6.6
Panonica NS	0.0	0.0	0.0	46.1	0.0	46.1	7.9
Lucija	0.0	0.0	3.9	0.0	46.1	48.7	1.9
Group 1	0.0	0.0	1.3	1.6	0.6	94.7	1.8
Group 2	0.0	0.0	0.9	0.4	0.0	1.5	95.8

Rows are reference and columns are predicted performance results (%). Group_1: Euclide, Soissions, Vulkan, Vulkanos, Angelos, SuperŽitark and Energo. Group 2: HYP, Cosinus, Mungles, Odisej and Ragetec.

The varieties were classified by k-NN, and as it can be seen in the confusion matrix some varieties were well classified (‘Agostino’ was classified with 97.4% accuracy), but many varieties such as ‘Vulkan’, ‘Angelus’ and ‘Vulkanus’ were classified correctly just with 15–27% accuracy. Table 1 shows the separation between the five best predicted varieties (Agostion, Calorius, Jana, Panonica NS and Lucija) and two groups of varieties with yellowish seeds that were sorted together in the blob plot according to the species (wheat/triticale) and size (short/long) of the seeds. The created groups show how classification accuracy of poorly sorted varieties, as individual ones, can be greatly improved. At the same time, with this pathway, was intended to indicate the gradual sorting procedure of almost indistinguishable varieties. To aid the complete separation of each variety by multispectral imaging, the use of a stepwise grouping classification method regarding suitable morphological and nCDA developed features is recommended according to the demonstrated results. In breeding new resistant varieties, this would be valuable information. With the use of hyperspectral imaging a 98.89% correct classification of maize varieties was reported [9] and likewise an overall 85.72% accuracy rate for sorting wheat varieties [18], confirming that in order to successfully discriminate varieties, a more suitable identifiable feature must be found, such as wavelength, color and texture

### SKNIR (Single Kernel Near-InfraRed spectroscopy)—chemometrics analysis

**Fig 5:**
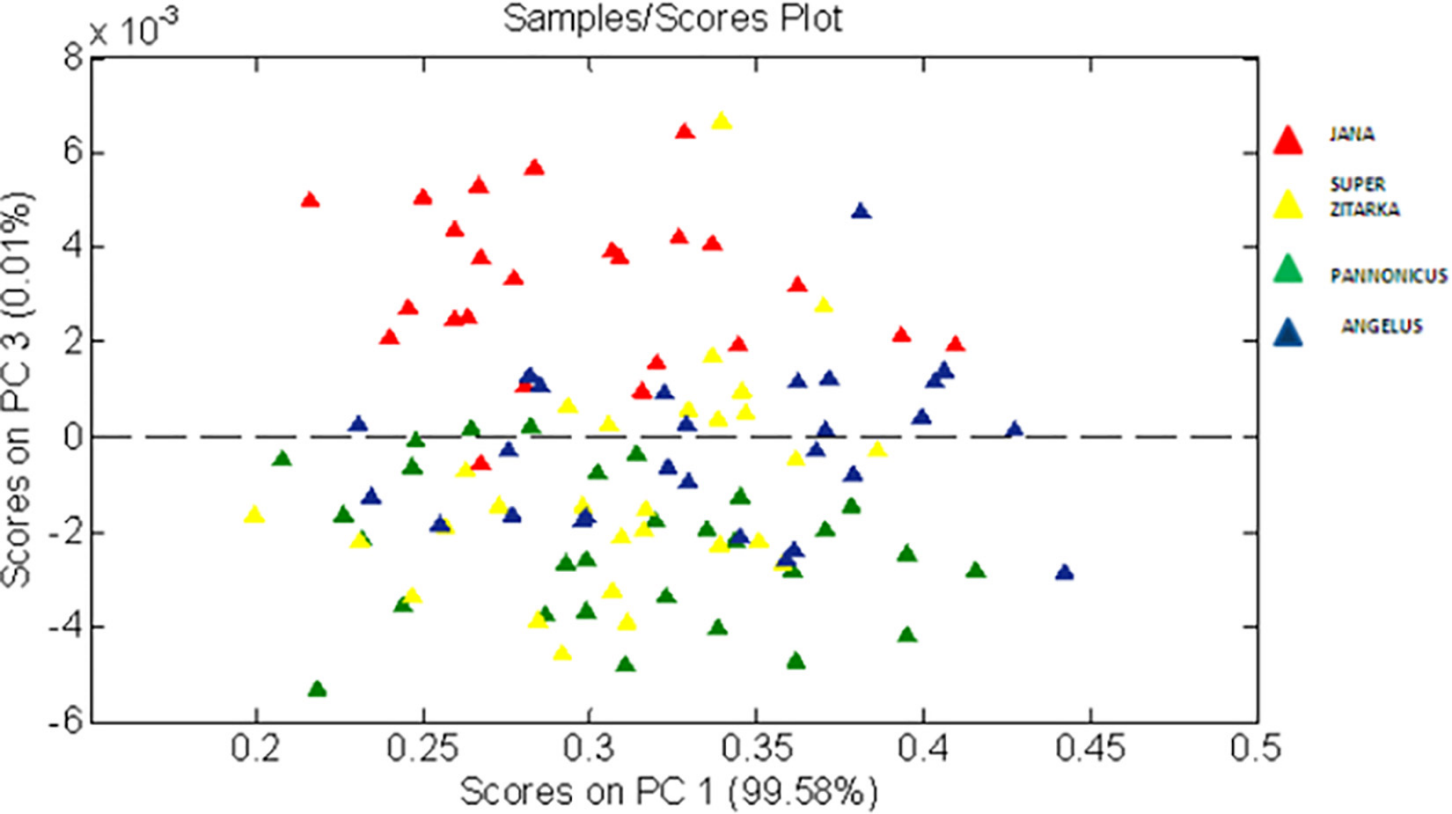
PCA plot of randomly chosen wheat varieties. PCA score plot on Jana (red), Angelus (blue), Super žitarka (yellow) and Pannonikus (green) where PC 3 shows a clear separation between Jana and Pannonikus and also between Jana and Angelus.

The principal component analysis on all 36 varieties showed no differences between varieties for principal component one (PC 1). Therefore, it was decided to make a PCA analysis on a reduced data set (Jana, Pannonikus, Super žitarka and Angelus varieties), shown on Fig 5. The PCA analysis on the reduced data set shows that Jana could be separated from the other varieties using PC3. PC1 explained 99.6%, PC2 0.04% and PC3 0.01% of the variation meaning that PC3 was still explaining a small percentage of the variation. The total variation explained by PC1 to 3 was close to 100%.

There are no clear peaks in the loading plot from PC3, which shows that the information from PC3 comes from different chemical constituents in the spectra. Therefore, it is difficult to relate information from PC3 to any specific chemical information. Thus, the preliminary conclusion is that the use of SKNIR needs further development in order to be able to identify more differences between varieties and explain why PCA can find these differences. The distance between lines could also present genetic variations [19] which might be useful information, especially in breeding for organic agriculture, where the focus is on high-diversity approaches [20]. Advances in variety classification by deoxynivalenol (DON) level produced by *F*. *culmorum* and *graminearum* might also be supplemented [21].

SKNIR has proved to be a potential automatic sorting device of healthy and Fusarium-damaged grains [22, 21]. Peiris et al. [23] expected that SKNIR as a non-destructive sorting device could provide information to breeders even for complicated issues as DON-content as on protein contains, hardness, virtuousness, kernel density, all important wheat quality characteristics connected with the genetic phenotypic expression [7, 13].

## Conclusion

According to the indicated results, multispectral imaging performed excellently in the separation between *Fusarium*-infected parts of the seeds from uninfected parts as well as for robust variety separations (color, size, species) with currently available software and nCDA features. The overall results on varieties separation with multispectral imaging shows that just one variety (Agostino) was classified with 97.4% of accuracy; for the rest of varieties classification accuracy was below 67%. Sorting accuracy could be greatly improved (up to 95.8%) in case of grouping varieties together according to visual similarity (color, size and species). Varieties classification with SKNIR performed poor results. Data did not show desired grouping. Much additional research and testing is needed in order to be able to develop an effective sorting model with non-destructive methods. The two methods, presented in this study, have perspective and non-research capacity for seed health and variety identification diagnostics. Therefore, study findings may provide great assistance for further research work. Once the models combining imaging and SKNIR could be designed and automatized, it could accelerate the seed health testing process, remove seed impurities and assist in selecting desired varieties in the breeding procedure as well as in the seed production industry.

## Supporting Information

S1 Fig Videometer Lab instrument (375nm-970nm) for capturing multispectral images. (PDF)

S2 Fig FT-NIR analyzer (900–1600 nm) used for SKNIR analysis. (PDF)
