# Supplementary figures and images for: The Use of Image-Spectroscopy Technology as a Diagnostic Method for Seed Health Testing and Variety Identification
Source: PLoS One. 2016 Mar 24;11(3):e0152011. doi: 10.1371/journal.pone.0152011 (PMC4807013; doi:10.1371/journal.pone.0152011)

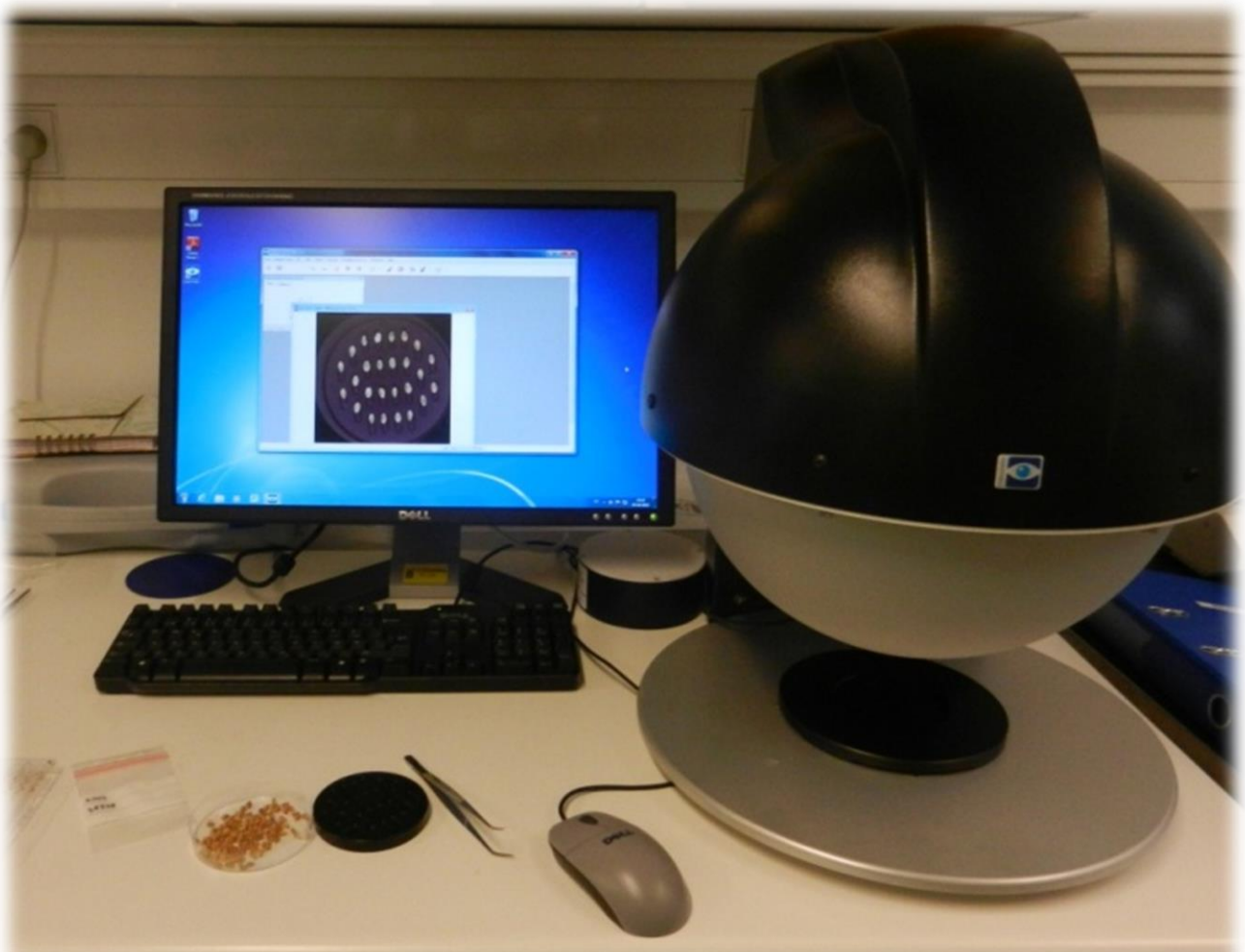

VIS

NIR

Supplement: S1 Fig — (PDF) [file pone.0152011.s001.pdf]

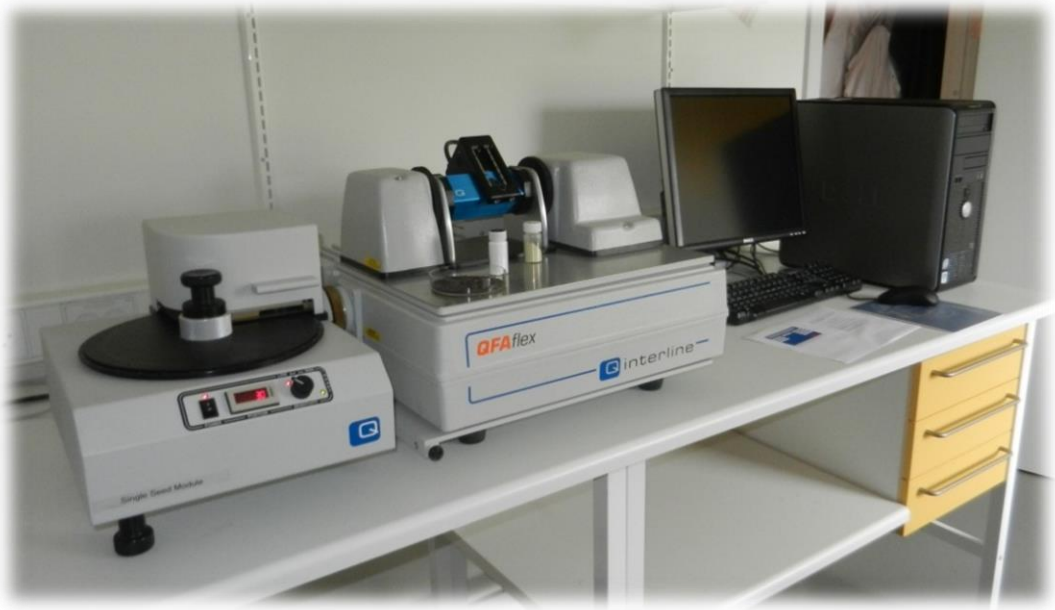

Supplement: S2 Fig — (PDF) [file pone.0152011.s002.pdf]
